# An Integrated Disease/Pharmacokinetic/Pharmacodynamic Model Suggests Improved Interleukin-21 Regimens Validated Prospectively for Mouse Solid Cancers

**DOI:** 10.1371/journal.pcbi.1002206

**Published:** 2011-09-29

**Authors:** Moran Elishmereni, Yuri Kheifetz, Henrik Søndergaard, Rune Viig Overgaard, Zvia Agur

**Affiliations:** 1 Institute for Medical Biomathematics (IMBM), Bene-Ataroth, Israel; 2 Novo Nordisk A/S, Malov, Denmark; 3 Optimata Ltd., Ramat-Gan, Israel; Max-Planck-Institut für Informatik, Germany

## Abstract

Interleukin (IL)-21 is an attractive antitumor agent with potent immunomodulatory functions. Yet thus far, the cytokine has yielded only partial responses in solid cancer patients, and conditions for beneficial IL-21 immunotherapy remain elusive. The current work aims to identify clinically-relevant IL-21 regimens with enhanced efficacy, based on mathematical modeling of long-term antitumor responses. For this purpose, pharmacokinetic (PK) and pharmacodynamic (PD) data were acquired from a preclinical study applying systemic IL-21 therapy in murine solid cancers. We developed an integrated disease/PK/PD model for the IL-21 anticancer response, and calibrated it using selected “training” data. The accuracy of the model was verified retrospectively under diverse IL-21 treatment settings, by comparing its predictions to independent “validation” data in melanoma and renal cell carcinoma-challenged mice (R^2^>0.90). Simulations of the verified model surfaced important therapeutic insights: (1) Fractionating the standard daily regimen (50 µg/dose) into a twice daily schedule (25 µg/dose) is advantageous, yielding a significantly lower tumor mass (45% decrease); (2) A low-dose (12 µg/day) regimen exerts a response similar to that obtained under the 50 µg/day treatment, suggestive of an equally efficacious dose with potentially reduced toxicity. Subsequent experiments in melanoma-bearing mice corroborated both of these predictions with high precision (R^2^>0.89), thus validating the model also prospectively *in vivo*. Thus, the confirmed PK/PD model rationalizes IL-21 therapy, and pinpoints improved clinically-feasible treatment schedules. Our analysis demonstrates the value of employing mathematical modeling and *in silico*-guided design of solid tumor immunotherapy in the clinic.

## Author Summary

Among the many potential drugs explored within the scope of cancer immunotherapy are selected cytokines which possess promising immune-boosting properties. Yet, the natural involvement of these proteins in multiple, often contradicting biological processes can complicate their use in the clinic. The cytokine interleukin (IL)-21 is no exception: while its strength as an anticancer agent has been established in several animal studies, response rates in melanoma and renal cell carcinoma patients remain low. To help guide the design of effective IL-21 therapy, we have developed a mathematical model that bridges between the complex biology of IL-21 and its optimal clinical use. Our model integrates data from preclinical studies under diverse IL-21 treatment settings, and was validated by extensive experiments in tumor-bearing mice. Model simulations predicted that beneficial, clinically practical IL-21 therapy should be composed of low-dose schedules, and/or schedules in which several partial doses are administered rather than a single complete dose. These findings were subsequently confirmed in mice with melanoma. Thus, future testing of these strategies in solid cancer patients can be a promising starting point for improving IL-21 therapy. Our model can thus provide a computational platform for rationalizing IL-21 regimens and streamlining its clinical development.

## Introduction

Cancer is a multi-faceted disease, involving complex interactions between neoplastic cells and the surrounding microenvironment [1]. The prospect of immunotherapy, i.e. stimulating endogenous immune responses by various molecular and cellular factors, is emerging as a promising approach against this disease [1], [2], [3]. One of the latest candidates for solid cancer immunotherapy is Interleukin (IL)-21, a γc-signaling protein of the IL-2 cytokine family with versatile immune-modulating properties [4], [5], . IL-21 has demonstrated substantial antitumor responses in several independent preclinical studies, in which mice inoculated with diverse transplantable syngeneic tumor lines were treated with the drug via cytokine-gene transfection, plasmid delivery, or injection of the recombinant protein [9]. In Phase I and IIa clinical trials, IL-21 was well tolerated and triggered moderate antitumor activity in some renal cell carcinoma (RCC) and metastatic melanoma (MM) patients [10], [11], [12], [13], [14]. More recently, clinical trials of IL-21 in combination with the tyrosine kinase inhibitor sorafinib for the treatment of RCC, and Rituximab for the treatment of non-Hodgkin's lymphoma, have also been investigated with encouraging results [15].

Yet, the intricate biology of IL-21 may set hurdles for its clinical development. Produced mainly by activated CD4+ T cells, IL-21 induces anticancer immunity predominantly by stimulation of natural killer cells (NKs) and/or cytotoxic T lymphocytes (CTLs) [4], [5], [6], [7]. The cytokine regulates various cellular and humoral pathways of immunity, and exerts conflicting stimulatory and inhibitory effects on several cell types [9], [16], [17]. Recent evidence for anti-angiogenic effects of IL-21 [18] further complicates its dynamical influence on the tumor microenvironment. Considering this biological complexity, traditional “trial-and-error” methodologies for clinical IL-21 therapy design are likely inefficient, and ought to be replaced by new guided approaches to maximize drug efficacy.

Rational and systematic planning of anticancer therapy may be directed by mathematical modeling and computer-aided analysis, which provides a better understanding of the involved dynamics. Over the past 25 years, mathematical modeling strategies have been applied in oncology-focused studies investigating tumor progression, angiogenesis and interactions with the immune system [19], [20], [21], [22], [23], [24]. Models for cytotoxic, cytostatic and cytokine-based direct and supportive cancer drugs have been introduced, with some being subsequently validated in preclinical and clinical settings [23], [25], [26], [27], [28], [29], [30], [31], [32], [33], [34], [35], [36]. These strategies have highlighted the importance of adequate selection of therapeutic regimens to achieve desired outcomes, by carrying out in-depth analysis of optimal times, dosages, and durations of treatment. Pharmacokinetic (PK) and pharmacodynamic (PD) modeling of anticancer agents can be particularly useful for clinical design of treatment [37],[38].

We have previously developed a mathematical model for the local dynamic effects of IL-21 on solid cancers. The model focused on interactions of IL-21 with NKs/CTLs, effector cytotoxicity against target cells, and immune memory, providing initial understanding of the optimal conditions for IL-21 gene therapy [39],[40].

Here, we have designed a new comprehensive PK/PD/disease model to predict clinically relevant scenarios of IL-21 treatment following intravenous (IV) subcutaneous (SC) or intraperitoneal (IP) administration in different cancer indications. The model forecasts long-term effects of the drug by integrating newly described PK/PD processes together with a disease model, based on our initial *in situ* model [39],[40]. This new combined model was retrospectively and prospectively validated by *in vivo* experiments in IL-21-treated mice bearing melanoma (B16) or renal cell carcinoma (RenCa). Model predictions provide substantial insights concerning adequate planning of systemic IL-21 therapy in solid cancers.

## Materials and Methods

### Ethics statement

All experiments were conducted according to Novo Nordisk principles for animal studies, as approved by the Danish National Ethics Committee on Experimental Animals, and in accordance with National Institute of Health guidelines for the care and use of laboratory animals.

### Experimental data

Data were collected from a published preclinical study in which mice bearing B16 and RenCa tumors were treated with IL-21 by various strategies [41]. Briefly, tumors were induced at day 0, and a daily (B16) or 3×/week (RenCa) IL-21 regimen (50 µg/dose) was applied SC or IP either at an “early” stage (day 3 in B16; day 7 in RenCa), or at a “late” stage (day 8 in B16; day 12 in RenCa) of tumor development. The tumor was measured several times until experiment termination. Data were available from additional unpublished dose-titration experiments in RenCa: IL-21 was given SC, 1× or 3×/week, and groups of mice (n = 6) were assigned a dose between 1-50 µg. The complete database was *a priori* divided into “training datasets” for model parameter estimation, and “validation datasets” for model verification.

In new prospective experiments designed to test model-suggested regimens, 7-8-week-old wild type C57BL/6 mice (Taconic Europe A/S, Denmark) were inoculated SC in the right flank with 1×10^5^ B16F0 melanoma cells (American Type Culture Collection (ATCC), CRL-6322) on day 0. Recombinant murine IL-21 (Novo Nordisk A/S, Denmark) or PBS was injected SC from day 3, when tumors were visible. IL-21 was given at 12 µg/day, 50 µg/day, or 25 µg twice a day, each group including n = 10 mice. Tumor volumes were calculated by the formula 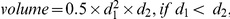
based on the two perpendicular diameters *d_1_* and *d_2_* measured approximately 3×/week with digital callipers. All experiments were carried out blindly, without the investigator's knowledge of model predictions. Animals were randomized and ear-tagged prior to treatment onset and euthanized when individual tumor volumes reached 1000 mm^3^.

### Model structure

**Figure 1:**
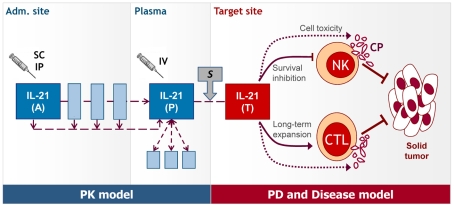
Scheme of the systemic IL-21 mathematical model. A model of IL-21 PD effects on immune regulation of tumor growth [39] is combined with a new IL-21 PK model based on data in mice [41]. Under SC/IP administration, IL-21 is introduced at site (A) and is transported through 3 compartments to the plasma (P). Under IV administration, the drug is injected directly into the plasma. The drug is degraded via 3 additional compartments. IL-21 concentrations in the target tissue (T) are correlated with the plasma levels by parameter *s*. In the target site, IL-21 inhibits NK survival and promotes CTL expansion, while enhancing CPs of both cells and facilitating their tumor cell targeting. Abbreviations: PK- pharmacokinetics; PD- pharmacodynamics; IV- intravenous; SC- subcutaneous; IP-intraperitoneal; NK- natural killer cell; CTL- cytotoxic T lymphocyte; CPs- cytotoxic proteins.

The new comprehensive systemic model for IL-21 immunotherapy contains PK/PD effects merged with disease interactions, as schemed in Fig. 1. The system is described hereafter, and the coupled ordinary differential equations (ODEs) are fully detailed in the Text S1 (sections A-B).

#### PK model

To describe IL-21 PK following standard administration routes, we used experimental profiles of IL-21 serum concentrations in mice after SC, IP, or IV application of a single 50 µg dose [41]. Since the PK events induced under IL-21 treatment are not fully defined, a non-traditional PK modeling strategy, involving generalized assumptions and a “multiple-modeling” approach, was employed. According to this approach several alternative PK models, differing in number of compartments and connectivity, were developed and tested, leading to the selection of the best performing one. The constructed models were all semi-physiological, incorporating standard PK processes (i.e. drug transport, absorption, and excretion). Each alternative structure was designed to support all three administration routes (SC, IP, and IV), and thus generalized to consider processes mutual or exclusive to the different administration routes. In addition, for every considered model structure and administration route, we calibrated not a single parameter set, but rather ten alternative sets that were tested for satisfying the PK model and fitting the data (for the detailed calibration process, see *Parameter estimation*). This approach, akin to similar multi-modeling strategies exercised in past comparable models [42],[43],[44], is thought to enhance the validity of the model: the predicted outcomes would not depend on one parameter set, and therefore would be more robust, and less sensitive to fluctuations [42].

The multiple-modeling approach identified a minimal, eight-compartment model, which effectively recreated the experimental IL-21 PK profiles under all three administration routes (Fig. 1, see Text S1, section A, for detailed equations and structure of the selected model). In the modeled IV injections, the drug is introduced directly to the plasma, from which it can be transported to three secondary tissues (e.g., the liver, kidneys and bile), reabsorbed to the plasma, degraded, or transferred into the target tissue. This four compartment IV model was chosen by analyzing the number of linearity regions in the data reported in [41] (see details in Text S1, section A). In SC and IP injections, the model contains continuous drug flow from the administration site, via up to three compartments, into the plasma site. The absorption structure was designed to allow: (1) participation of multiple peripheral compartments in IL-21 transition and decay; (2) non-sequential transition between compartments, i.e. multi-directional flow of the drug between tissues. This flexibility was motivated by our assumption that, similarly to other recombinant cytokines with complex PK profiles [45], IL-21 can potentially be taken up, cleared or processed, by several tissues and cells expressing its receptor. Indeed, the murine biodistribution profile in SC/IP administrations demonstrated observable multiplex transport of IL-21 into several peripheral tissues, where its concentrations were higher than plasma levels during several time points (personal communication, Dr. P. Thygesen, Novo Nordisk A/S, Denmark). Of note, similar multi-compartmental active transport was also described in prior models of IL-21-induced hematological effects in primates [46],[47], in support of this structure.

Non-linear Michaelis-Menten kinetics were initially assumed for all transition and degradation processes in the model. Such dynamics have been previously suggested as more suitable for cytokine models, since they are expected to be better at capturing complex drug disposition patterns [45]. We also reasoned that non-linearity would allow higher flexibility of the model and account for saturation-containing effects. Conversely, an a-priori linear assumption may cause bias towards underestimation or overestimation of some PK processes. Of note, non-linear dynamics also display clear advantages over simpler linear models in describing certain oncotherapies, and afford substantially improved goodness-of-fit to PK data [44]. Thus, in our model, the transition rate of IL-21 from any compartment *i* to any compartment *j* is given by the non-linear term *J_i,j_:*

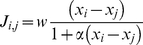
(1)


The latter is a rate-limiting function of the amounts *x_i_* and *x_j_,* where parameter *w* is a constant that always regulates the reaction rate and *α* determines the saturation level. The degradation rate of the drug from any compartment *i* in the model is described by term *D_i_*, a similar non-linear function of the amount *x_i_* (where *d* regulates the reaction rate and *β* determines the saturation level):
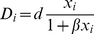
(2)


Selected drug transition and degradation events in the resulting eight ODEs model did in fact necessitate non-linear dynamics, as implied by values of the saturation PK parameters *α* and *β* (Text S1, Table S1): While the IP route may have been satisfied by a linear transport description (as evident by small and negligible *α* values), SC administration required a combination of linear and non-linear transport events (as *α* values were significant). Likewise, nonlinearity surfaced in some degradation processes under all administration routes (as shown by large values for *β*).

Finally, in the absence of sufficient experimental data on the rate of IL-21 transfer from plasma to the tissue, we assumed a direct correlation between tissue concentration and plasma concentrations. Thus, to relate IL-21 concentrations in the plasma (*x_0_*) to those in the target tissue (*x_T_*), the two compartments were correlated by a parameter, *s*. Drug levels at the tumor site are therefore a fraction of systemic levels at any given moment: 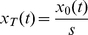
(3)


#### PD and disease model

In the target tissue, IL-21 achieves an anticancer response by modulating several immune components [4],[5],[6],[7]. The key *in situ* processes affected by the drug were described by our previous six ODEs model, which had successfully recreated the antitumor effects in mice subject to IL-21-gene therapy [39],[40]. The model comprised the IL-21-modulated dynamics of the immune effector cells NKs and CTLs, the intracellular cytotoxic proteins by which these effectors lyse the tumor cells, IL-21-induced memory ensuring a long-term CTL response, and the tumor growth (see Text S1, section B).

For the current PD/disease structure (Fig. 1), NK, CTL, cytotoxic protein, and memory factor populations were described as in the prior tissue model [39],[40]. However, some entities were altered herein: (1) IL-21 dynamics in the tissue were modified from the previous system, and set to be correlated with plasma IL-21 levels, effectively binding PK to PD (see above). (2) The new PD model assumes a CTL-dominance (rather than the prior equal effector balance) in the IL-21-mediated response (see *Parameter estimation*). This is based on the notion that CTLs are likely more influential than NKs in the scenario of systemic IL-21 treatment [41]. (3) To describe the baseline growth of both B16 and RenCa, the disease model here assumes a logistic function. (4) Parameter values of all prior components were adapted as needed, in order to comply with the systemic therapy settings, and with the new RenCa tumor analyzed herein (see *Parameter estimation*).

### Parameter estimation

The model (Fig. 1) was implemented in C (Microsoft Visual Studio.NET) and MATLAB (The MathWorks, Natick, MA) programming platforms. The system was solved by fourth-order Runge Kutta integration. Model parameters were evaluated by a customized numerical method based on Hooke and Jeeves optimization [48] combining global and local search heuristics and least-squares curve-fitting. Parameter sets achieving maximal model agreement with experimental training data were selected (see Text S1, Tables S1-S2).

#### PK parameters

For calibration of the diverse PK models considered in the multiple-modeling process, serum IL-21 concentrations following a single SC, IP, or IV injection in mice [41] were used as training datasets. Before calibration, the plasma volume was set at 2 ml and the injected dose at 50 µg. Each potential PK model was fit in a process that yielded ten possible parameter sets. The model was then simulated with these ten sets under diverse treatment settings: A model was deemed reliable when its multiple predictions (generated under all sets) were unified, under all therapeutic scenarios that were simulated. Thus, the final PK model selected for further simulation (see above, as also schemed in Fig. S1, and discussed in Text S1, section A) fulfilled this criterion. For each of the three administration routes accounted for in this PK model, one representative parameter set (of the ten sets) is displayed (see Table S1 in Text S1), and the respective fits are shown as well (Fig. S2A). The best-fitted PK model (Fig. S1) was selected for further simulation.

**Figure 2:**
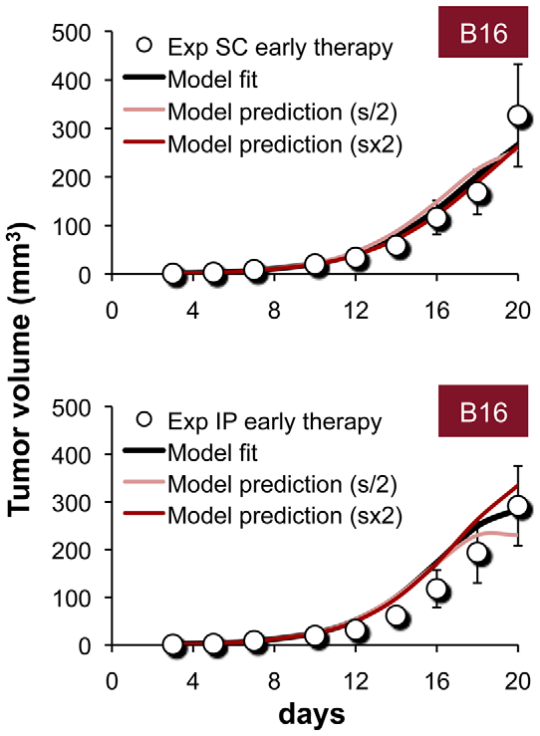
Estimation and sensitivity analysis of model parameter *s*. Curve-fits produced during estimation of parameter *s*, using experimental training data of B16 dynamics under an early-onset (day 3) IL-21 treatment (50 µg/day) [41]. The parameter was evaluated per route of administration (see Table S1 in Text S1), and model-data approximation is indicated for both SC and IP treatment (“Model fit”). Predictions of the model under 2-fold increased or decreased *s* values (“Model prediction s×2” and “Model prediction s/2”, respectively) retrieving these experimental data (Exp), are plotted. Simulations (lines) are shown with respect to data (circles), given as means±SEM.

Parameter *s,* relating tissue concentrations of IL-21 to the plasma concentrations of the drug, was estimated after evaluating all other PK/PD model parameters. Since tissue biodistribution data following SC vs. IP injections of IL-21 in unchallenged mice show different profiles ([41] and personal communication, Dr. P. Thygesen, Novo Nordisk A/S, Denmark), we allowed *s* to be administration-dependent by estimating this parameter separately for SC and IP. An initial value range for parameter *s* was obtained by comparing the drug profile in the blood to the profiles in various peripheral tissues, which provided a rough estimate of the ratio of blood:tissue drug concentrations at any given time. This gave a realistic range of *s* between 1–100. Exact estimation of *s* was accomplished by curve-fitting to training data of B16 dynamics following IL-21 treatment via an early-initiated (day 3) 50 µg/day SC/IP regimen [41] (see Fig. 2, “Model fit”). Final *s* values (Text S1, Table S1) were all within the predetermined biologically acceptable range.

#### PD and disease model parameters

Some parameters of the PD model component were set at their previous values ([39],[40], Table S2 in Text S1): Immune system parameters were set identical for both tumor types, as they reflect indication-independent processes (i.e. effector cell dynamics, cytotoxicity effects, and immune memory). Parameters *σ* and *D,* quantifying maximal CTL numbers, were taken as tumor-specific since they represent the immune response intensity and depend on the tumor immunogenicity [39]; their values for B16 and RenCa were set as in non-immunogenic and moderately immunogenic tumors, respectively [39].

Coefficients *k_1_* and *k_2_* (the respective NK and CTL interaction affinities to the tumor) were re-estimated to reflect a stronger influence for CTLs in the antitumor response (i.e. *k_1_*<*k_2_*), as suggested in these conditions [41]. To evaluate *k_1_* and *k_2_*, we used training data from B16-bearing mice in which these immune cells were neutralized prior to IL-21 immunotherapy [41]: *k_1_* was evaluated by curve-fitting to tumor dynamics in T-cell neutralized mice, thus assuming no CTL activity (i.e. *k_2_* = 0, Fig. S2B); *k_2_* was estimated using data from NK-neutralized mice (setting *k_1_* = 0) representing a lack of NK activity (Fig. S2B). The obtained *k_2_* value was larger than *k_1_* by one order of magnitude (Table S2 in Text S1), fulfilling the condition for CTL-dominance over NK. (Selected simulations were also performed using the original *k_1_* and *k_2_* values ensuing an equal tumor-killing role, i.e. *k_1_* = *k_2_*, which is appropriate in early tumorigenesis; see Text S1, section C).

B16 and RenCa growth parameters (Table S2 in Text S1) were newly estimated using training data from control (PBS-treated) mice [41]. Model-evaluated tumor cell numbers were scaled to volume (mm^3^) units, assuming that 10^6^ cells equal 1 mm^3^ [39]. Estimations were carried out by curve-fitting to data from early treatment conditions where tumors are small at therapy onset (Fig. S2C), and from late treatments consisting of large initial tumors (data not shown). Thus, diverse growth parameter values were obtained for each tumor type and therapeutic onset.

### Model simulation and validation

The model was simulated under numerous IL-21 regimens, differing in onset, duration, dose, inter-dosing interval, route, etc. All simulations were repeated several times to ensure output consistency. Retrospective verification of the model was accomplished by checking its prediction accuracy, via statistical comparison of its output with prior independent validation datasets (see *Experimental data* and [41]): Model simulations were conducted under the specific tumor settings and treatment conditions of each prior experiment. For prospective model validation, selected model-identified regimens were tested experimentally, and results were statistically compared to model predictions at the data sampling times.

### Statistical analysis

The goodness-of-fit between the model output and experimental data was determined by calculating the coefficient of variation (*R^2^*). To compare between experimental datasets, Student's t-test (two-tailed, assuming equal variance) was applied. A *P*<.05 value was considered statistically significant.

## Results

### Sensitivity analysis of model parameters

First, we examined the sensitivity of the model to small variations in the value of the plasma-tissue correlation factor *s*, being that this pivotal parameter simplifies rather complex PK processes. Simulations of the experimental early-onset IL-21 regimen (50 µg/day applied SC/IP) in the B16-challenged setting were carried out under diverse *s* values, in the vicinity of those obtained through curve-fitting (see *Materials and methods* and Fig. 2). After increasing or decreasing *s* values by two-fold, model predictions still accurately retrieved the murine data (R^2^>0.90; Fig. 2), and were comparable to the original fits (Fig. 2, “Model fit”). Interestingly, model predictions remained precise even when modifying the values of the effector-tumor interaction coefficients *k_1_* and *k_2_* (see Text S1, section C, and also Fig. S3). These results indicate that model predictions are robust even when *s, k_1_* and *k_2_* values slightly diverge, meaning that different numeric combinations of these parameters, i.e., multiple NK:CTL ratios, can accomplish the same therapeutic effect. This implies a potentially wide window of IL-21 doses within which effects may be comparable.

### Retrospective model validation by experiments in IL-21-treated mice with B16 tumors

**Figure 3:**
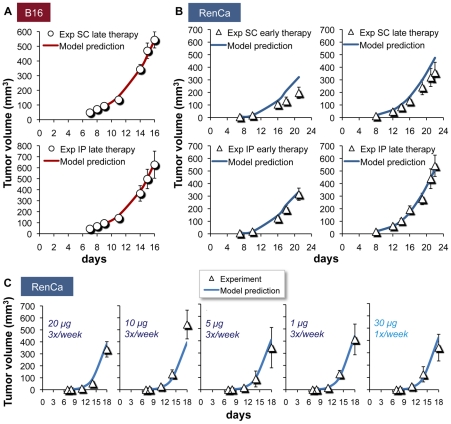
IL-21-induced antitumor effects: model simulations retrospectively verified in experimental murine tumors. Model predictions (lines) retrieve experimental validation data (circles, triangles) of tumor dynamics from a preclinical study [41], where (A) B16-bearing mice were treated by a 50 µg/day IL-21 treatment applied SC or IP, starting on day 8 after tumor inoculation; (B) RenCa-challenged mice were treated by IL-21, 50 µg 3×/week, SC or IP, commencing either early (day 7) or late (day 12) after tumor inoculation; (C) RenCa-bearing mice were SC-administered various IL-21 doses between 1–20 µg (3×/week), or given a 30 µg (1×/week) regimen. Data are given as means±SEM.

Our primary goal was to validate the model's predictive accuracy. We therefore compared its output to the experimental B16 progression following a late (day 8) onset regimen of IL-21, given at 50 µg/day SC/IP for 3 weeks [41]. All late treatment simulations were strongly in line with the independent validation data (R^2^>0.90; Fig. 3A), thus verifying the model. Notably, the model was able to recapitulate the biological behavior even under the aforementioned modifications in *s*, *k_1_* and *k_2_* parameter values (data not shown).

### Retrospective model validation by experiments in IL-21-treated mice with RenCa tumors

Next we assessed the model's generality by investigating whether it can predict IL-21 therapy outcomes in other solid cancer indications, such as RenCa. Tumor growth and selected immune system parameters were set for RenCa, using training data in untreated mice and previously calibrated parameter values for moderately-immunogenic cancers (see *Materials and methods* and [39]). Other parameter values were set exactly as in the B16 case. Simulations of the experimentally-applied IL-21 treatment of 50 µg at 3×/week, given for 3 weeks [41], showed model predictions to be strongly akin to the observed dynamics under late (day 12) therapy administered SC, as well as in early (day 7) and late IP regimens (R^2^>0.90; Fig. 3B). Under the early SC regimen, predicted responses were slightly weaker than observed, yet still remained within the measurement's standard deviation (R^2^>0.73; Fig. 3B, upper panel).

To further validate the model for RenCa, we simulated it to predict the effects another experiment that applied lower IL-21 doses (between 1–20 µg, SC 3×/week for 3 weeks). Predictions were in agreement with the validation set readouts in most doses (R^2^>0.94; Fig. 3C), collectively demonstrating a moderate dose-dependent decrease in IL-21-mediated tumor eradication. The 10 µg (3×/week) simulation experiment gave a good, but slightly lower, model-data correlation (R^2^>0.83; Fig. 3C). The model also successfully retrieved a retrospective experiment testing a 30 µg (1×/week) IL-21 treatment schedule (R^2^>0.90; Fig. 3C).

### Improved model-based IL-21 regimens and their prospective validation in B16-challenged mice

**Figure 4:**
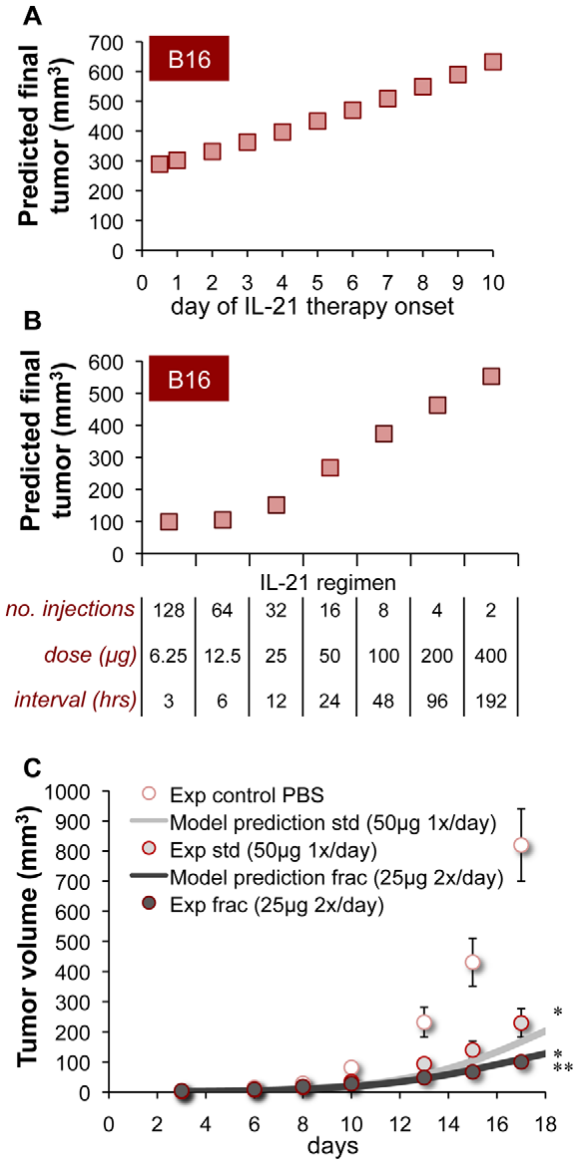
Model-improved IL-21 therapies with modified onset and fractionation. (A) Predicted outcomes (final B16 volumes; squares) of 20-day regimens (50 µg/day given SC) initiated on different days. (B) Predicted outcomes of regimens with the same total IL-21 dose (800 µg/treatment given SC) yet with different fractionations (i.e. number of injections, inter-dosing intervals and dose intensities). (C) Prospective validation of the model predictions (lines) in B16-bearing mice treated by a standard (std) 50 µg/day regimen vs. a fractionated (frac) 25 µg/twice daily schedule, both administered SC between days 3–20 (data in circles). Tumor growth in PBS controls is indicated as well. Means±SEM of data are given (n = 10; *p<0.001 for 25 µg-treated mice vs. PBS-treated mice, and for 50 µg-treated mice vs. PBS-treated mice; **p<0.05 for 25 µg-treated mice vs. 50 µg-treated mice).

Having validated the model, we used it to gain insights into better IL-21 therapy in the B16 setting. In particular, we searched for regimens that would be superior to the standard daily SC 50 µg treatment applied previously [41]. First, we tested whether the treatment initiation time is a critical factor in determining IL-21 effects, by simulating different onsets of the standard daily regimen. The model predicted that earlier therapy initiation results in stronger anticancer responses, as expected (Fig. 4A). The simulated tumor mass at the end of therapy (day 20) was lowest under the earliest regimen, which began one day after B16 challenge: This final tumor load was roughly 15% lower than that obtained in the standard treatment initiated at day 3. In contrast to this early regimen, the tumor load resulting from a delayed regimen, initiated at day 10, was doubled (Fig. 4A). Further delayed regimens (with onsets as high as day 17) were even less favorable (data not shown). These results collectively emphasize the importance of early-onset therapies. Notably, however, not even the earliest treatment onset was able to fully eradicate the tumor.

Simulations were performed also to see whether the anticancer response could be improved by fractionating the IL-21 regimen into a more intensive high-dosing protocol, as suggested for other drugs [34],[49]. To design alternative schedules, the daily IL-21 regimen (16 SC injections, 50 µg each, given from day 3; [41]) was taken as a reference point: the same total dose (800 µg) was distributed differently across the treatment window, using various doses and inter-dosing intervals, creating a collection of regimens to be tested. Intriguingly, the model predicted that a more intensive schedule, applying two 25 µg doses per day at a 12-hour inter-dosing interval, would lead to a 45% lower tumor mass than that obtained under the standard daily 50 µg regimen (Fig. 4B). Fractionation into even smaller doses given every few hours produced slightly lower tumor sizes, yet these responses were not significantly better than the 25 µg regimen outcomes (Fig. 4B). In fact, not even the most fractionated schedule could arrive at full eradication of the tumor. At the other end, less fractionated regimens comprising large IL-21 doses given every few days had significantly weaker efficacy (Fig. 4B).

In order to verify our prediction that the fractionated 25 µg/12 hour regimen would be superior to the standard 50 µg/24 hour schedule, the two were experimentally applied in B16-challenged mice. Even though both schedules effectively attenuated tumor progression as compared to control PBS-treated mice (*p<.001; Fig. 4C), the 25 µg/12 hour regimen was considerably more successful than the standard 50 µg daily regimen (**p<.05; Fig 4C), as mathematically predicted. The observed tumor dynamics under the 25 µg regimen had an excellent fit with the prior model predictions (R^2^>0.90; Fig. 4C), providing strong and quantitative prospective validation of the model's precision.

**Figure 5:**
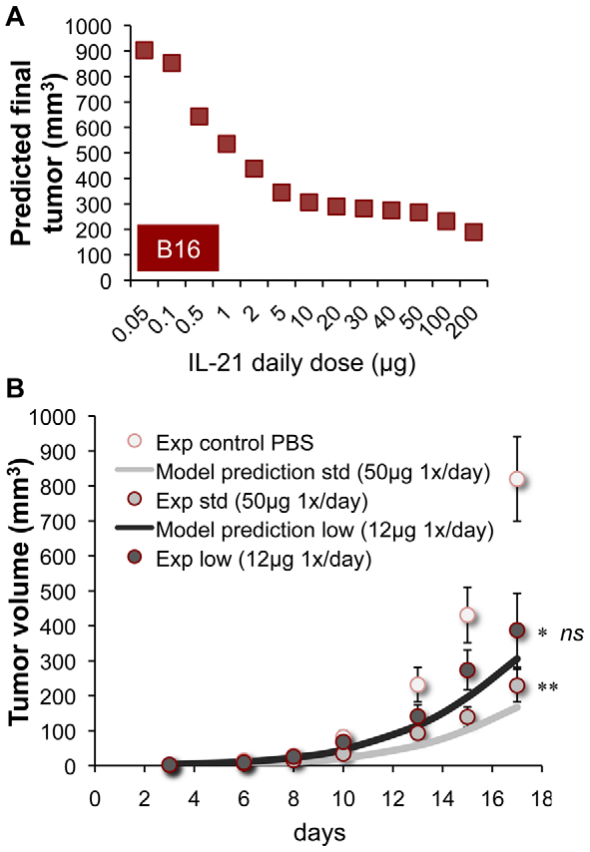
Model-improved alternative-dosing IL-21 regimens. (A) Predicted outcomes (final B16 volumes) of various 20-day treatments (initialized at day 3, and given SC), where different daily dose are applied (squares). (B) Experimental B16 dynamics following prospective treatments under the standard (std) 50 µg/day regimen, or under a model-based reduced-dosing (low) schedule (12 µg/day). Data (circles) are shown vs. model simulations (lines). Tumor growth in PBS controls appears as well. Means±SEM of data are indicated (n = 10; *p<0.05 for 12 µg-treated mice vs. PBS-treated mice; **p<0.001 for 50 µg-treated mice vs. PBS-treated mice; ns-not significant for 12 µg-treated mice vs. 50 µg -treated mice).

We considered that the fractionated regimen may not be clinically practical, since it could involve increased costs of therapy, and, at least in IV delivery, would possibly require hospitalizing patients. Therefore, the search for better treatment was limited to simple, widely-acceptable daily administration schedules. Regimens of one IL-21 dose per day (e.g. 16 SC injections given between days 3–20 following B16 inoculation) were simulated under different dose intensities: A dose-dependent increase in the response, reflected by lowered tumor masses, was predicted for very low (<5 µg) or very high (>50 µg) levels (Fig. 5A). Yet interestingly, similar outcomes were predicted for the 5–50 µg dose range (Fig. 5A). This might be explained by the conflicting roles of IL-21, enhancing CTL activation while drastically reducing NK numbers at the same time [39]; It is likely that in this dosing range, IL-21-increased CTL responses fail to promote further tumor shrinkage due to the IL-21-inhibition of NK availability.

A prospective experiment in B16-induced mice examined whether a low dosing regimen in the plateau range (i.e. 12 µg/day) could indeed be as effective as the standard 50 µg/day treatment. Beginning on day 3 following tumor challenge, the two doses were applied SC, and the tumor mass was measured until day 17. Both the 12 µg and 50 µg doses induced sufficient antitumor responses in the mice (*p<.05 and **p<.001 compared with PBS-treated mice; Fig. 5B). Although the 12 µg dose appeared slightly less potent, its effect was not significantly different from the 50 µg schedule (ns, p>.05; Fig. 5B), as anticipated by the model. Indeed, the model prediction (Fig. 5A) fit the 12 µg/day outcome to a good degree (R^2^ = 0.89, Fig. 5B). These findings further validate the model, and are the first indication of an IL-21 dosing range executing equally potent effects.

## Discussion

Immune-targeted therapy is increasingly apparent in the battle against cancer. Several reagents are in development within this scope, some already approved for use in certain indications [1],[9],[50]. In this study, we have devised and validated a clinically-relevant mathematical model integrating the PK/PD effects on immune and disease interactions of IL-21, one of the recent immunotherapeutic drugs under focus in solid cancers [9]. Following its verification, our model was used for suggesting beneficial IL-21 treatment policies.

Previous attempts to model cytokine-based immune modulation of solid malignancies have been mainly theoretical, helping to elucidate certain characteristics of the tumor-immune system cross-talk and providing important insights into treatment success (see for example [23],[31],[33]). Our former model focused on the heart of the IL-21 response, retrieving the effects of cytokine gene therapy to a good extent. Yet, its predictions could not be extrapolated to the clinical realm. The current work is thus among the first biomathematical studies accounting for practical treatment aspects of cytokine immunotherapy in general, and IL-21 treatment in particular. Our current model deals with realistic PK and PD effects on disease progression, clinically-feasible scheduling, patient compliance, etc. Moreover, in contrast to the customary stand-alone PK/PD modeling approach, we have integrated IL-21 PK/PD with specific effects on the involved biological processes, to give a mechanistic, yet minimal, model. Particularly, our PD/disease model accounts for real entities of the IL-21 biological processes (effector cells, etc.), which enabled us to use measurable data and make testable quantitative predictions. At the same time, we kept our model concise thanks to condensation of other overly complex biological entities (cytotoxic proteins, etc.) which are less cardinal and often not measured experimentally. Overall, our approach provides a robust model that can forecast the long-term anticancer effects of a specific immunotherapeutic cytokine, via a clinically-oriented prism.

Our integrated PK/PD model was constructed by an advanced “multiple-modeling” approach, which we found most suitable for the IL-21 scenario. The selection of a favorable model out of many analyzed structures and complexities, and the use of non-linear kinetics, enabled us to explore significantly more functional possibilities, and allowed for flexibility in the design. Moreover, rather than forming a model per scenario, we were able to create a generalized model by describing processes that are mutual to different therapeutic settings (administration routes, etc.) and tumor types. This enhanced the robustness of the model, since it structure was subject to testing under diverse conditions. Indeed, the model encompasses IL-21-induced outcomes in a wide range of treatment conditions, under different times and administration routes. Despite its simplicity, the model accurately predicted IL-21-relayed effects in B16- and RenCa-challenged mice, both prospectively and retrospectively. Moreover, the model demonstrated robust behavior, and predictions were largely insensitive to modulation of key parameters. With this combined generality and accuracy, the model can potentially accommodate other clinical settings and solid cancers where similar immune processes apply and where IL-21 has been useful (i.e. adenocarcinoma, glioma, neuroblastoma) [7],[8].

A systematic design of clinically applicable IL-21 immunotherapy strategies has long been called for. Considering the modest responses of MM and RCC patients to IL-21 therapy [10],[11],[12],[13],[14], it is worthwhile to examine whether the drug can be more powerful under different treatment approaches. Previous trial regimens of IL-21 were determined based on the US Food and Drug Administration guidelines for high-dose IL-2 therapy in MM patients [13], as the two cytokines share homology and certain effector-inducing functions. Yet, recent findings demonstrate that IL-2 and IL-21 do not entirely align in their actions [17],[51],[52], inferring that the optimal administration strategies (administration routes, dose intensities, inter-dosing intervals, etc.) likely vary between the two agents. Local IL-21 delivery or expression have been proposed, by us and others, to be potentially effective and safe approaches [17],[39],[53], yet such therapeutic methods are not yet available for clinical use. Our systemic model analysis therefore represents a new effort to identify improved, clinically-appropriate IL-21 therapies, using the preclinical tumor models B16 and RenCa as case studies.

Simulations of differently dosed IL-21 schedules gave rise to central new insights. According to the model, comparable antitumor responses are induced by daily IL-21 doses within the 5–50 µg range. This was prospectively confirmed in B16-challenged mice, in which a substantially lower IL-21 dose (roughly 12 µg/day) was as effective as the standard 50 µg/day treatment. An insensitive range of IL-21 doses with similar efficacy is not unreasonable, considering that the drug respectively inhibits or induces NKs and CTLs, two cells which complement one another in the process of cancer targeting. This model-aided identification of smaller doses with similar therapeutic efficacy could have immense clinical value, possibly reducing putative IL-21-associated toxicities. Adverse events have indeed been reported in IL-21-treated patients [10],[11],[12],[13],[14]. IL-2 and interferon-α, other cytokine drugs, are associated with severe hematological and neuropsychiatric side effects complicating their use [2]. Recent PK/PD models of toxic IL-21 effects on body temperature and red blood cell regulation [46],[47] present a possible framework in which our improved regimens can be confirmed for clinical safety.

Another interesting concept surfacing from our simulations addresses IL-21 fractionation. The model predicted improved antitumor responses by simple partitioning of the experimental regimen (a single 50 µg dose/day) into an equally intense regimen of 25 µg doses given twice daily. This was prospectively validated by experiments in which the fractionation-treated mice ended therapy with ca. half of the tumor load observed after the standard treatment. Model-predicted halving of a daily dose was sufficient to significantly enhance IL-21 efficacy, and further division of the doses was not imperative. Indeed, fractionation of cancer therapeutics was recommended in the past by mathematical modeling [29], and its beneficial effects have been validated preclinically for a chemotherapy supportive drug [34]. This strategy has mostly been applied in the context of radiation therapy and chemotherapy [54], yet our results, which clearly indicate the benefit of fractionated IL-21 dosing, propose its relevance also to immune-modulating drugs. Notwithstanding, fractionation may be impractical, reducing patient compliance and requiring hospitalization in certain cases. Moreover, embarking on new clinical studies to test fractionation therapy is a large and expensive task, and further adjustment of the mathematical model to humans is needed before engaging in such endeavors. Our findings also raise the question whether IL-21 ought to be administered by available “slow and continuous release” drug delivery methods, which can be viewed as regimens of maximal partitioning. Past cytokine-gene therapy experiments in mice showed complete eradication of IL-21-secreting tumors in which the drug was released in low continuous levels directly in the target tissue [7],[9], supporting the possible advantage of fractionated regimens. Future implementation of such routes of drug delivery within our model can allow to specifically analyze the benefit of such strategies for IL-21 therapy.

Our present results set the stage for constructing a humanized IL-21 model, to serve as a tool for streamlining development of the drug, and in the future, hopefully, also for personalizing cytokine immunotherapy. The model, up-scaled to the clinical arena, can entertain diverse cancer indications, patient-specific characteristics, and different modes of therapy. Newly-discovered IL-21 properties of relevance to the anticancer response, such as modulation of T regulatory cell functions [17] and anti-angiogenic properties [18], may be introduced in the evolving IL-21 model. Finally, considering the growing interest in combination therapies for solid cancers, and the promising preclinical and clinical responses observed when applying IL-21 with monoclonal antibodies or signaling inhibitors [9],[50], a future model will also study IL-21 therapy in combination with additional therapeutic reagents.

## Supporting Information

Figure S1 **Scheme of the IL-21 PK model.** The PK model consists of 4 compartments for IV administration, or of 8 compartments for SC/IP drug application. IL-21 dynamics in each compartment (denoted by *x*) are mathematically detailed in Section A. Parameters *k* regulate drug transfer rates, and parameters *d* control drug degradation rates.
(TIF)

Figure S2 **Model curve-fitting for evaluation of selected parameters.** Parameters newly introduced in the systemic PK/PD model were estimated by curve-fitting, according to the data in [1]. (A) Final model fits following the calibration of PK parameters, which utilized data from normal healthy mice that were IL-21 administered (50 µg) via IV, SC, and IP routes. (B) Fits obtained in the evaluation of NK and CTL affinity parameters, by data from diseased IL-21-treated mice in which CTLs and NKs were neutralized (respectively). (C) Fits obtained in the evaluation of B16 and RenCa growth parameters, via data from diseased untreated (control) mice. Means±SEM of data are indicated.
(TIF)

Figure S3 **Sensitivity analysis of effector-tumor interaction parameters.** Retrieval of experimental training data of B16 dynamics under early IL-21 treatment (50 µg/day), by the model, assuming either a “CTL-dominating” response (*k_1_*<*k_2_*; see parameter estimation in *Materials and methods*), or an “equal NK/CTL balance” response (*k_1_* = *k_2_*) inspired by the previous gene-therapy model [2],[3]. Simulations (lines) are shown with respect to data (circles), given as means±SEM.
(TIF)

Text S1 **Detailed description of PK model equations (section A), PD and disease model equations (section B), analysis of parameter sensitivity (section C), and parameter values for the full PK/PD/disease model (Tables S1-S2).**
(DOC)
