# Supplementary figures and images for: An Integrated Disease/Pharmacokinetic/Pharmacodynamic Model Suggests Improved Interleukin-21 Regimens Validated Prospectively for Mouse Solid Cancers
Source: PLoS Comput Biol. 2011 Sep 29;7(9):e1002206. doi: 10.1371/journal.pcbi.1002206 (PMC3182868; doi:10.1371/journal.pcbi.1002206)

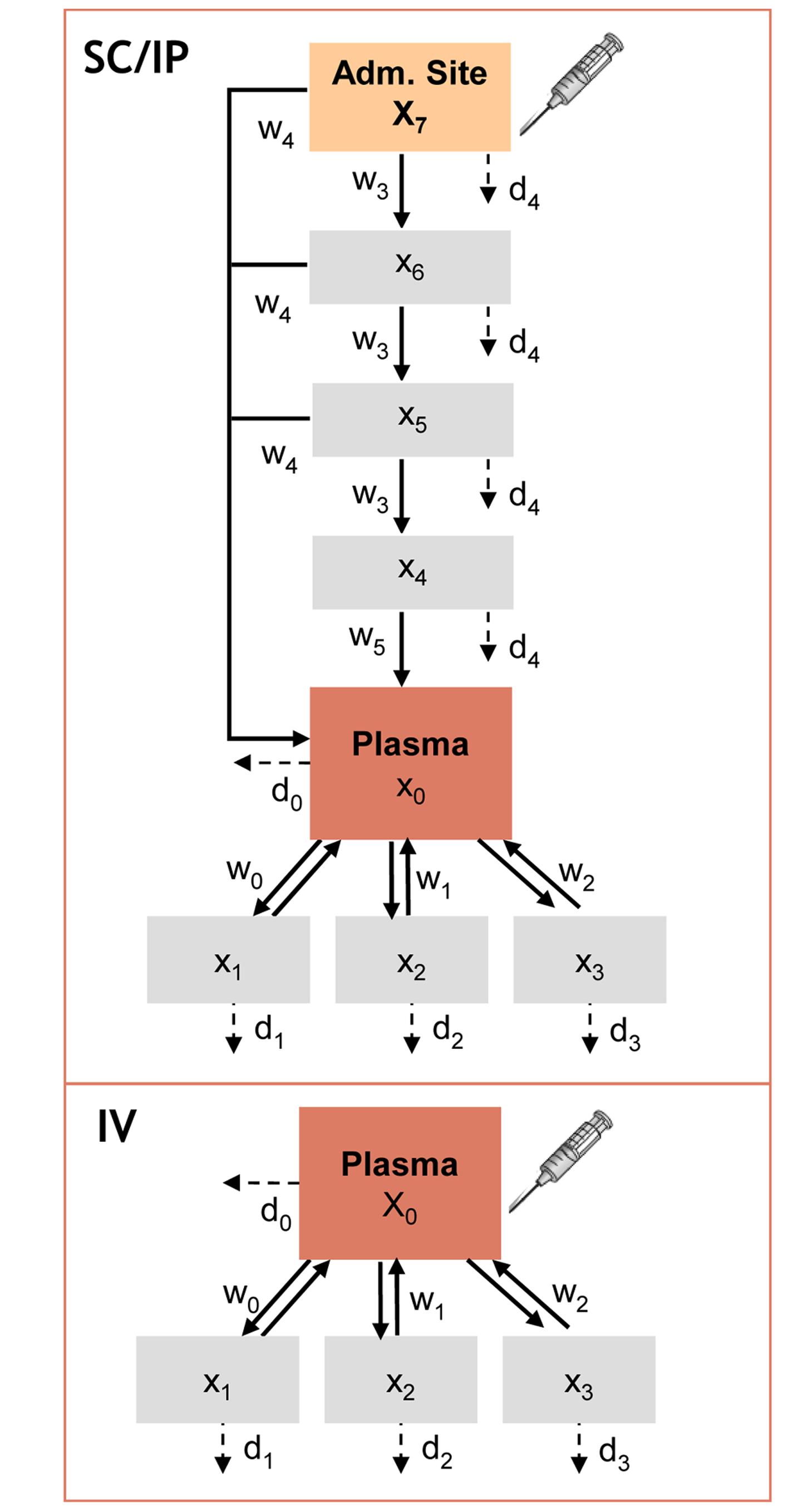

Supplement: Figure S1 — Scheme of the IL-21 PK model. The PK model consists of 4 compartments for IV administration, or of 8 compartments for SC/IP drug application. IL-21 dynamics in each compartment (denoted by x) are mathematically detailed in Section A. Parameters k regulate drug transfer rates, and parameters d control drug degradation rates. (TIF) [file pcbi.1002206.s001.tif]

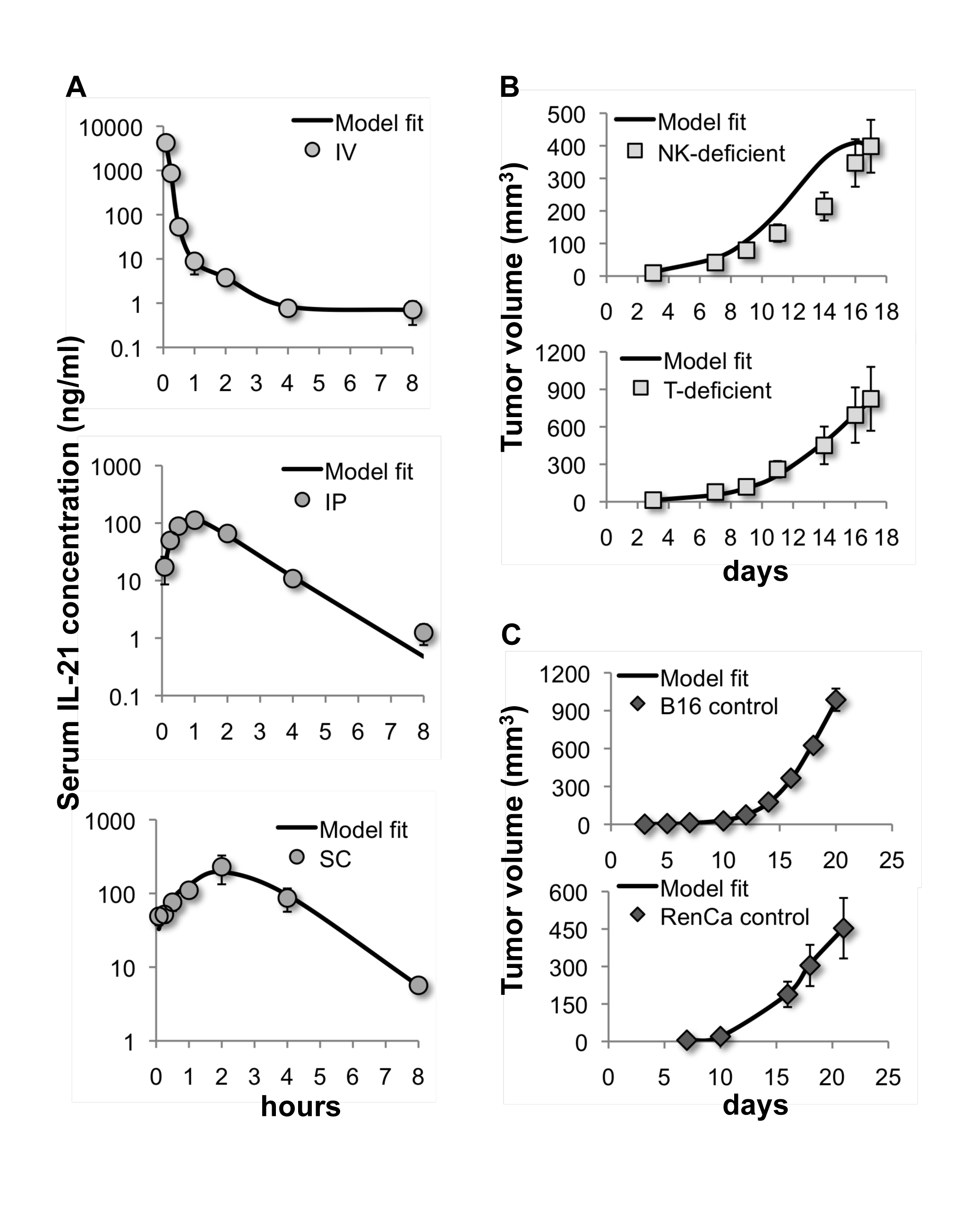

Supplement: Figure S2 — Model curve-fitting for evaluation of selected parameters. Parameters newly introduced in the systemic PK/PD model were estimated by curve-fitting, according to the data in [1]. (A) Final model fits following the calibration of PK parameters, which utilized data from normal healthy mice that were IL-21 administered (50 µg) via IV, SC, and IP routes. (B) Fits obtained in the evaluation of NK and CTL affinity parameters, by data from diseased IL-21-treated mice in which CTLs and NKs were neutralized (respectively). (C) Fits obtained in the evaluation of B16 and RenCa growth parameters, via data from diseased untreated (control) mice. Means±SEM of data are indicated. (TIF) [file pcbi.1002206.s002.tif]

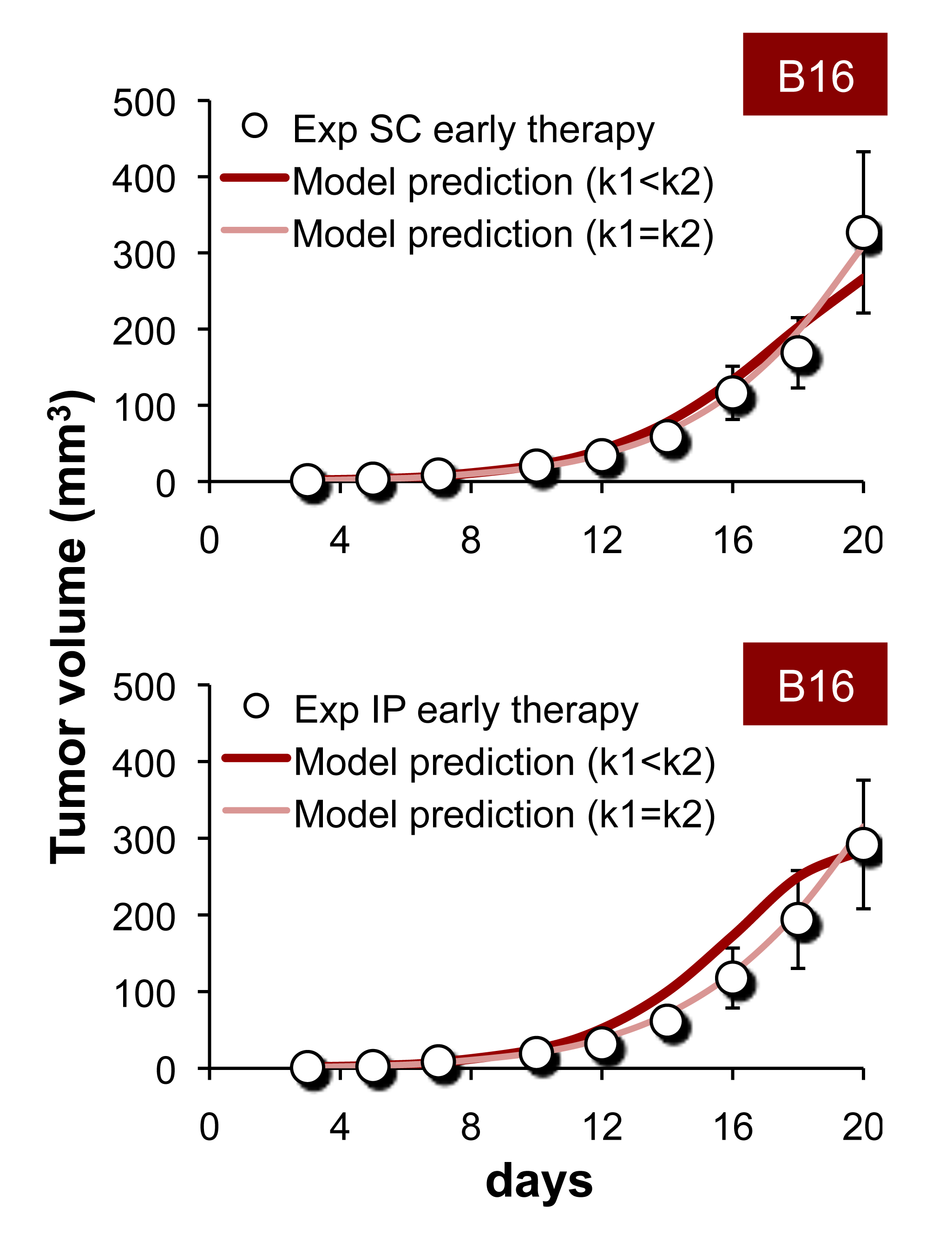

Supplement: Figure S3 — Sensitivity analysis of effector-tumor interaction parameters. Retrieval of experimental training data of B16 dynamics under early IL-21 treatment (50 µg/day), by the model, assuming either a “CTL-dominating” response (k1<k2; see parameter estimation in Materials and methods), or an “equal NK/CTL balance” response (k1 = k2) inspired by the previous gene-therapy model [2],[3]. Simulations (lines) are shown with respect to data (circles), given as means±SEM. (TIF) [file pcbi.1002206.s003.tif]
