# Supplementary material for: An Integrated Disease/Pharmacokinetic/Pharmacodynamic Model Suggests Improved Interleukin-21 Regimens Validated Prospectively for Mouse Solid Cancers
Source: PLoS Comput Biol. 2011 Sep 29;7(9):e1002206. doi: 10.1371/journal.pcbi.1002206 (PMC3182868; doi:10.1371/journal.pcbi.1002206)
Supplement: Text S1 — Detailed description of PK model equations (section A), PD and disease model equations (section B), analysis of parameter sensitivity (section C), and parameter values for the full PK/PD/disease model (Tables S1-S2). (DOC) [file pcbi.1002206.s004.doc]

**Text S1**

**A. PK model equations**

The non-linear eight-compartment PK model devised herein (Fig. S1) consists of a central plasma compartment (*x_0_*), which is the first target of drug application in the case of IV administration. Thereon, the drug influxes into three additional compartments (*x_1_*-*x_3_*) that allow for degradation and re-absorption processes. Our initial selection of this 4-compartment model was based on data reflecting IL-21 serum kinetics after IV administration [1]. Particularly, we assessed the dependence of *ln(x(t))* on *t,* where *x(t)* is the drug concentration and *t* is the time. We approximated *ln(x(t))* with a linear piecewise function, and the number of linearity regions suggested the number of compartments required. The data [1] consisted of 7 time points (with 2-3 values, i.e. mice, per each time point), and we performed naïve pooling, i.e. assuming the data comes from one mouse. Using Student’s t-test, we found substantial differences (p-value < 0.05) between slopes of different line segments. Four linear regions were detected, indicative of 4 compartments. Of note, subsequent simulations supported this strategy, as models with less than 4 compartments did not yield a satisfactory fit to the data.

For SC and IP administration routes, four compartments (*x_4_*-*x_7_*) precede the plasma compartment (*x_0_*): the drug is applied at the injection site (*x_7_*) and is actively transported to the plasma (*x_0_*) through up to three compartments (*x_4_*-*x_6_*), enabling continuous multiscale drug flow. The dynamics in these model compartments are thus given by the following equations (Eq. S1-S9):

(Eq. S1);

(Eq. S2);

(Eq. S3);

(Eq. S4);

(Eq. S5);

(Eq. S6);

(Eq. S7);

(Eq. S8);

(Eq. S9);

All processes of drug transfer and degradation were initially formulated with non-linear sigmoid dynamics. Drug decay is regulated by coefficients *d*, and transfer between compartments is controlled by coefficients *w*. Parameters denoted *V_i_* convey the volumes of compartment *x_i_*, and parameters denoted *α_i_* and *β_i_* enable non-linearity of the described processes. All components are initialized at 0. At time (*τ*), when the dose D is applied, compartments receiving the drug at the dose denoted D are assigned the value of

$x_{0}\left(\tau\right)=\frac{D}{V_{0}}$ (Eq. S10),

$x_{7}\left(\tau\right)=\frac{D}{V_{4}}$ (Eq. S11),

for IV and SC/IP routes, respectively.

Tissue-localized IL-21 concentrations (*x_T_*) were correlated with systemic concentrations, by setting the drug concentration *in situ* as a fraction of the concentration in the plasma (*x_0_*) at any given time point *t*:

$x_{T}(t)=\frac{x_{0}(t)}{s}$ (Eq. S12).

Thus, parameter *s* is responsible for the simple linear correlation between these two compartments, and is assumed to be unique for each administration route.

All parameters were estimated through the multiple-modeling approach, and using data from [1], by curve-fitting (as shown in Fig. S2A herein, and in Fig. 2A). For each administration route, ten alternative sets were estimated, and a representative parameter set is provided in Table S1. See detailed procedures in *Materials and methods*.

**B. PD and disease model equations**

Our model for the antitumor effects of IL-21 in solid tumors, adapted from our previous work [2, 3], includes six essential components in the target tissue: IL-21 concentrations (*x_T_*), NKs (*n*), specific CTLs (*c*), an element facilitating CTL long term ‘memory’ effects (*m*), cytotoxic proteins mediating tumor lysis (*p*), and finally the tumor cells (*z*). In our current PD model, IL-21 dynamics in the tissue are directly correlated with those in the plasma (Eq. S11). As in [2, 3], the rest of the components are structurally given by the following system of ordinary differential equations (Eq. S13-S19 respectively):

(Eq. S13),

(Eq. S14),

(Eq. S15),

(Eq. S16),

(Eq. S17).

The NK cell carrying capacity (Eq. S13) is influenced in a decreasing saturated manner:

$h_{1}\left(x_{T}\right)=\frac{p_{1}x_{T}+p_{2}}{x_{T}+q_{1}}$, (Eq. S18),

and the carrying capacity of specific CTLs (Eq. S14) is an increasing saturated function of the memory factor *m*:

$h_{2}\left(m\right)=h_{2}\left(0\right)+\frac{\sigma m}{1+\frac{m}{D}}$ (Eq. S19).

The tumor growth law (in Eq. S17) was set to be logistic for both tumor types (B16 and RenCa), as was used for the B16 tumor line in our initial study [2, 3]. Some parameters were set at their prior values, while others were newly estimated by curve fitting with new data from [1], as shown in Fig. S2, B-C (see *Materials and methods*). Values and units are listed in Table S2.

**C. Analysis of parameter sensitivity: *k_1_* and *k_2_***

The current IL-21 model was simulated mostly under CTL-dominating conditions, appropriate for systemic treatment in established tumors [1]; *k_2_* was set to be 10-fold higher than *k_1_* (see *Materials and methods*). Yet, we recognized that the tumor-effector cell interaction affinity is, in reality, more complex, and that the NK/CTL balance is subject to change under diverse tumor stages and therapeutic conditions. Indeed, our prior “gene-therapy” model was validated under the assumption that NKs and CTLs are of equal significance (*k_1_* = *k_2_* = 0.5), better reflecting processes in early tumor development and therapy [2, 3]. To assess whether the current systemic model is sensitive to different NK/CTL balances, we performed simulations not only in the *k_1_* < *k_2_* setting, but also under diverse *k_1_* and *k_2_* values. Particularly, the *k_1_* = *k_2_* scenario of similar NK/CTL importance (our original assumption; [2, 3]) was tested. Intriguingly, model predictions under both conditions (e.g. equal NK/CTL balance vs. CTL dominance) were comparable (Fig. S3), as they equally retrieved IL-21-responses in B16/RenCa-bearing mice under the early regimen (50 µg/day applied SC/IP). These data imply that the systemic model is indifferent to changes in the NK/CTL balance, further demonstrating its generality.

**Tables**

**Table S1.** PK parameter values.

| **Parameter** | **SC** | **IP** | **IV** | | **Units** |
| --- | --- | --- | --- | --- | --- |
| *V_0_* | 2.0 | | | | ml |
| *V_1_* | 1.994 | | | |
| *V_2_* | 19.026 | | | |
| *V_3_* | 1.104 | | | |
| *V_4_* | 30.055 | 29.139 | na | |
| *w_0_* | 49.823 | | | | hours^-1^ |
| *w_1_* | 0.44 | | | |
| *w_2_* | 0.428 | | | |
| *w_3_* | 8.886 | 4.943 | | na |
| *w_4_* | 2.51 | 0.271 | | na |
| *w_5_* | 49.895 | 3.85 | | na |
| *d_0_* | 18.828 | | | | hours^-1^ |
| *d_1_* | 9.658 | | | |
| *d_2_* | <0.001 | | | |
| *d_3_* | 16.766 | | | |
| *d_4_* | 0.184 | 0.551 | | na |
| *α_0_* | <0.001 | | | | (ng/ml)^-1^ |
| *α_1_* | <0.001 | | | |
| *α_2_* | <0.001 | | | |
| *α_3_* | 0.0194 | 0.00167 | | na |
| *a_4_* | 0.0118 | 0.00283 | | na |
| *a_5_* | 0.734 | <0.001 | | na |
| *β_0_* | <0.001 | | | | (ng/ml)^-1^ |
| *β_1_* | 0.00240 | | | |
| *β_2_* | 930.285 | | | |
| *β_3_* | 0.242 | | | |
| *β_4_* | 0.00516 | <0.001 | | na |
| *s* | 11.92 | 1.065 | | na | ND |

na- not applicable. ND- non-dimensional.

**Table S2.** PD and disease model parameter values.

| **Parameter** | **Tumor type** | | **Units** |
| --- | --- | --- | --- |
|  | ***B16*** | ***RenCa*** |  |
| *r*_1_ | 0.095 | | days^-1^ |
| *p*_1_ | 0.01 | | cells·ml·ng^-1^· 10^6^ |
| *p*_2_ | 1.054 | | cells· 10^6^ |
| *q*_1_ | 0.54 | | cells· 10^6^ |
| *a* | 0.57 | | ng^-1^· ml· days^-1^ |
| *μ*_2_ | 1.4·10^-2^ | | days^-1^ |
| *r*_2_ | 0.26 | | days^-1^ |
| *h*_2_*(0)* | 0.0018 | | cells· 10^6^ |
| *σ* | 0.0071 | 0.003 | cells· 10^6^ |
| *D* | 0.19·10^3^ | 1.4·10^3^ | non-dimensional |
| *b*_1_ | 0.1 | | days^-1^· ng· ml^-1^ |
| *b*_2_ | 0.1 | | ng·ml^-1^ |
| *μ*_3_ | 0.08 | | days^-1^ |
| *z(0)*^*^ | 0.382 | 0.223 | cells· 10^6^ |
| *z(0)*^**^ | 4.114 | 1.599 | cells· 10^6^ |
| *r*_3_^*^ | 0.018 | 0.018 | days^-1^ |
| *r*_3_ ^**^ | 0.014 | 0.012 | days^-1^ |
| *K* ^*^ | 1501.4 | 541.7 | cells· 10^6^ |
| *K* ^**^ | 4688.1 | 1438 | cells· 10^6^ |
| *k*_1_ | 0.376 | | days^-1^· cells^-2^· ng^-1^· ml |
| *k*_2_ | 5.184 | | days^-1^· cells^-2^· ng^-1^· ml |

^*^ Values estimated based on control tumor dynamics in early treatment.

^**^ Values estimated based on control tumor dynamics in late treatment.

**References**

1. Sondergaard, H., et al., *Interleukin 21 therapy increases the density of tumor infiltrating CD8(+)T cells and inhibits the growth of syngeneic tumors.* Cancer Immunol Immunother, 2007.

2. Cappuccio, A., M. Elishmereni, and Z. Agur, *Cancer immunotherapy by interleukin-21: potential treatment strategies evaluated in a mathematical model.* Cancer Res, 2006. 66(14): p. 7293-300.

3. Cappuccio, A., M. Elishmereni, and Z. Agur, *Optimization of interleukin-21 immunotherapeutic strategies.* J Theor Biol, 2007. 248(2): p. 259-66.

4. Agur, Z., R. Hassin, and S. Levi, *Optimizing chemotherapy scheduling using local search heuristics.* Operations Research, 2006. 54(5): p. 829-846.
